# Halogen Bond via an Electrophilic π-Hole on Halogen in Molecules: Does It Exist?

**DOI:** 10.3390/ijms25094587

**Published:** 2024-04-23

**Authors:** Pradeep R. Varadwaj

**Affiliations:** 1 Department of Chemical System Engineering, School of Engineering, The University of Tokyo, 7-3-1, Tokyo 113-8656, Japan; pradeep@t.okayama-u.ac.jp; 2 Molecular Sciences Institute, School of Chemistry, University of the Witwatersrand, Johannesburg 2050, South Africa

**Keywords:** Halogen’s π- and σ-holes, π-hole halogen bond, σ-hole halogen bond, crystallography, intermolecular geometries and energies, MESP, QTAIM, IGM, NBO, SAPT-based characterizations

## Abstract

This study reveals a new non-covalent interaction called a π-hole halogen bond, which is directional and potentially non-linear compared to its sister analog (σ-hole halogen bond). A π-hole is shown here to be observed on the surface of halogen in halogenated molecules, which can be tempered to display the aptness to form a π-hole halogen bond with a series of electron density-rich sites (Lewis bases) hosted individually by 32 other partner molecules. The [MP2/aug-cc-pVTZ] level characteristics of the π-hole halogen bonds in 33 binary complexes obtained from the charge density approaches (quantum theory of intramolecular atoms, molecular electrostatic surface potential, independent gradient model (IGM-*δg^inter^*)), intermolecular geometries and energies, and second-order hyperconjugative charge transfer analyses are discussed, which are similar to other non-covalent interactions. That a π-hole can be observed on halogen in halogenated molecules is substantiated by experimentally reported crystals documented in the Cambridge Crystal Structure Database. The importance of the π-hole halogen bond in the design and growth of chemical systems in synthetic chemistry, crystallography, and crystal engineering is yet to be fully explicated.

## 1. Introduction

A plethora of self-assembled crystal shapes, synthetically developed through the remarkable adhesion engineering of non-covalent interactions, have been widely discussed and cataloged in the Cambridge Structural Database (CSD) [1,2,3] and Inorganic Crystal Structure Database (ICSD) [4,5,6], among others [7]. Halogen bond (or HaB for short) [8], which bears a striking resemblance to hydrogen bonding [9] and other non-covalent interactions [7,10,11], stands out as a prominent subset of non-covalent interactions. It was coined around 1978 [12], vigorously came to light after 2003 [13,14], and was integrated into the broader class of σ-hole interactions after 2007 [15]. Halogen in molecular entities can form close contacts with atoms in another species with which it interacts, which can be seen in studies that have appeared since the late 1990s in the gas phase [16] and since the mid-eighteenth century in crystals [17,18,19]. Its dynamical bonding aspect was elusive until Odd Hassel was awarded the Nobel Prize in Chemistry in 1969 for recognizing its fundamental importance in the understanding of charge transfer phenomena in complexes [20].

Halogen bonding [8], regardless of its strength, develops in a chemical system when there is evidence of a net attractive interaction between an electron-density deficient electrophilic region on the electrostatic surface of a halogen atom in a molecular entity and a close-lying electron-density rich nucleophilic region on the electrostatic surface of the same or another identical or different molecular entity [21]. A number of its translucent caveats and features appeared in the non-covalent chemistry literature may be suitable for the recognition of halogen bonding in chemical systems. The IUPAC definition of the halogen bond [8] mirrors the definitions of the hydrogen bond [9], chalcogen bond [7], and pnictogen bond [10,22]; the only difference between the former and the latter three is that the terms such as “halogen” and “halogen bond” in the former were replaced by “hydrogen, or chalcogen, or pnictogen” and “hydrogen bond, or chalcogen bond, or pnictogen bond” in the latter. In other words, the definition of the halogen bond [8] is nothing but a writing variant of the definition of the hydrogen bond [9], with the term “hydrogen” in the latter being replaced by “halogen” in the former. The study that proposed the definition of the halogen bond has narrowed down a number of accompanying features and notes recommended for hydrogen and chalcogen bonds, yet all these definitions are essentially based on the same underlying concept: “An electrophile on a covalently bonded halogen/hydrogen/chalcogen/pnictogen in a molecular entity attracts a nucleophile on the same/another molecular entity”. The base of the concept is clearly Coulombic.

Studies have shown that HaB is electrostatically driven [23,24,25]. Contributions arising from the exchange–repulsion, polarization, and dispersion interactions meticulously play an important role in explaining the net binding energy of HaB in HaB-driven complexes [25,26,27,28,29]. Arguments in support of an orbital-based charge transfer interaction that drives the formation of halogen bonds have also been developed and appeared in journals in a timely manner [30,31,32,33]. The debate continues, focusing largely on the similarities and differences between the role played by polarization and charge transfer [31,34,35], emerging from the blooming of Nobel Laureate Odd Hassel’s chemistry [35]. Additionally, it is commonly believed that halogen bonds are highly directional [36,37], with the R–X···Y (X = halogen; Y = nucleophile; R = remainder part of the molecule R–X) bond angle being typically close to 180° [35], and the linearity of the halogen bond has been argued to be better explained by charge–transfer interactions and lone pair repulsion [38,39]. As shown in this study, the view is narrow. This is because halogen bonds can potentially be nonlinear depending on the mode of the interaction of covalently bonded halogen with the nucleophile; thereby, the underlying refulgent phenomena of halogen bonding are yet to be fully appreciated [40].

The key to recognizing HaBs in molecules or crystal systems is to identify the halogen bond acceptors (HaBAs) and halogen bond donors (HaBDs) that interact due to attractive forces toward the molecular assembly. Halogen bond acceptors are locally nucleophiles (Lewis bases) (viz. N-ends of N_2_), and HaBDs are locally electrophiles (Lewis acids) (viz. X-ends of X_2_ (X = F, Cl, Br, I)). Clearly, the rudimentary force that combines the HaBA and HaBD to shape a crystal is a result of the net attraction driven by their opposite charge capacities, in line with the IUPAC definition of the HaB [8] and our revisit to the definition [21]. Insight into the formation of HaBs can also be revealed in terms of the conceptual theoretical framework of Pearson’s hard and soft acids and bases (HSAB) [41,42], corroborating the importance of the fundamental Coulomb’s law in electrostatics in that opposite charges on interacting atomic/molecular entities can attract each other when in close proximity.

In a simple chemical system, such as the hydrogen fluoride (HF) molecule, it may be immediately axiomatic that H and F have opposite capacities of charge (H^δ+^ and F^δ−^), and the halogen-end of the molecule cannot be a HaBD [43]. As the number of atoms that make up an arbitrary molecule increases, it is not very straightforward to readily capture the complicated distribution of charge density profile on the surfaces of the constituent atomic basins. This is because the distribution of charge density is generally anisotropic on the surface of atoms in a molecular entity; some parts of covalently bonded atom X in R–X may have insufficient electron density, while other positions of the same atom may have abundant electron density. This means that the electrophilicity or nucleophilicity of a region on X in R–X is determined not only by the electrons and nuclei in that region but also by the electrons and nuclei in the rest of the molecule, especially those in neighboring portions. If the electron density deficiency on atom X is sufficiently large, then atom X has what is commonly referred to as carrying a “hole” and may have a positive charge capacity (δ^+^) locally and is, therefore, electrophilic. Such electrophilic regions have been identified in large numbers on the side of X opposite to the covalently bonded halogen derivatives in molecular entities. They have been called positive σ-holes (pronounced “sigma-hole”) [15,44,45], or even referred to as electrophilic caps [46], and are prone to form HaBs when placed in close proximity to nucleophiles on other identical or different partner molecules.

The name “σ-hole” emerges from the notion that charge density deficiency on the covalently bonded atom X appears along the extension of the σ covalent bond, but, literally, it has nothing to do with a “hole” (the charge density deficiency is the “hole”) [17]. A negative σ-hole can be found on an atom X in R–X when it locally carries a negative charge capacity [47], as on F in H–F [48], H_3_C–F [49,50], and C_6_H_5_F [47]; it is not prone to HaB with a nucleophile on another identical or different interacting molecule. However, it can host itself as a nucleophile for an electrophilic σ-hole on halogen on another molecule when in close proximity, resulting in the formation of a σ-hole halogen bond. Thousands of intensive studies have been reported to identify and characterize halogen bonds formed by electrophilic σ-holes on halogen derivatives in molecules. When halogen atoms in molecules are hypervalent, they may host themselves as biaxial (σ-hole) halogen bond donors [28,51]. However, the electrophilicity of a π-hole on the halogen derivative in molecular entities and the diversity of its halogen bond-forming ability have not been carefully addressed; this is likely to be the subject of intense research for the coming decade.

A π-hole is a region of abundant or deficient electron density on the surface of a covalently bonded atom, or array of atoms, that is orthogonal to the bonding direction, or plane, of the molecule. Therefore, a π-hole can be either electrophilic or nucleophilic [47,52,53]. While π-holes correspond to a flattening of the electronic density surface, they are craters above and below the rings in benzene derivatives and in polyazines [52]. For example, the π-holes on the electrostatic surface of either side of the centroid of the C_6_ carbon ring of benzene and fluorobenzene (C_6_H_6_ [54] and C_6_H_5_F [47], respectively) are nucleophilic and, thus, are referred to as negative π-holes [47,53]. If all six hydrogen atoms of benzene are replaced by six fluorine atoms, positive π-holes are developed above and below the ring, and thus, C_6_F_6_ has two positive π-holes [47,53]. Similarly, both HCCH and N_2_ have triple bonds, which were conventionally understood as electron-rich in the C≡C and N≡N internuclear regions. However, the C≡C bonding region in H-C≡C-H has a negative belt-like π-hole, while the N≡N bonding region in N_2_ has a positive belt-like π-hole [55,56].

A recent study has demonstrated that π-holes in molecules can be visualized by means of Kelvin probe force microscopy [57], although the chemical binding capacity of negative π-holes has been known over several decades [29,58] and that of positive π-holes on main group elements has only begun to be studied within the current decade [59,60,61]. It was argued that if both electrostatics and polarization are taken into account, counterintuitive interactions involving π-holes can be treated as Coulombic [58,62], despite the apparent repulsion between ground-state molecules [34,37]. Opposite views have also been advanced [27,63]. The most commonly observed chemical systems where both σ- and π-holes are integrated are halogenated aromatic compounds.

Does an electrophilic π-hole exist on the surface of halogen derivative in molecules? Does it form a halogen bond when it is in the vicinity of a nucleophile on the partner molecule with which it interacts? To our knowledge, this question has not been properly addressed before, and the answer to both questions is “yes, it does exist” and “capable of halogen bond”. This is demonstrated in this paper with examples of crystals from CSD [1,2,3], known for many years, in which the π-hole interactions in them were neither named nor properly characterized nor assigned as π-hole halogen bonds. To this end, we have placed our focus on some simplified halogenated chemical systems that radiate π-holes on their electrostatic surfaces. We reveal this based on the application of the MP2 level of theory and the molecular electrostatic surface potential (MESP) tool to the XY_3_ (X = Cl, Br; Y = F, Cl, Br) set of molecules. We then demonstrate whether the electrophilic π-hole (Lewis acid), for instance, in BrCl_3,_ is capable of forming π-hole halogen bonds when interacting with a series of nucleophiles (Lewis bases) hosted individually by 32 other partner molecules and the power of chemical reactiveness of the π-hole on covalently bonded halogen as π-hole halogen bond donors. We have utilized the quantum theory of atoms in molecules (QTAIM) [64,65,66] and the Independent Gradient Model (IGM-*δg^inter^*) [67,68] approaches to characterize the π-hole halogen bonds, including a discussion of their energy strengths and geometric signatures. A higher-order symmetry-adapted perturbation theory (SAPT2+(CCD)) [69,70] was also applied to provide insight into the nature of dissected energy components that may explain the dominant factors responsible for the interaction energies of the π-hole halogen bonded complexes investigated, where the many-body treatment of dispersion was based on coupled-cluster doubles (CCD) [71]. The NBO’s [72] second-order perturbative estimates of ‘donor–acceptor’ (bond–antibond) interactions in the NBO basis [73] were discussed for some complexes to evince the possibility of hyper-conjugative charge transfer interactions between interacting monomers.

## 2. Results and Discussion

### 2.1. π–Hole Halogen Bond Donor Molecules

**Figure 1:**
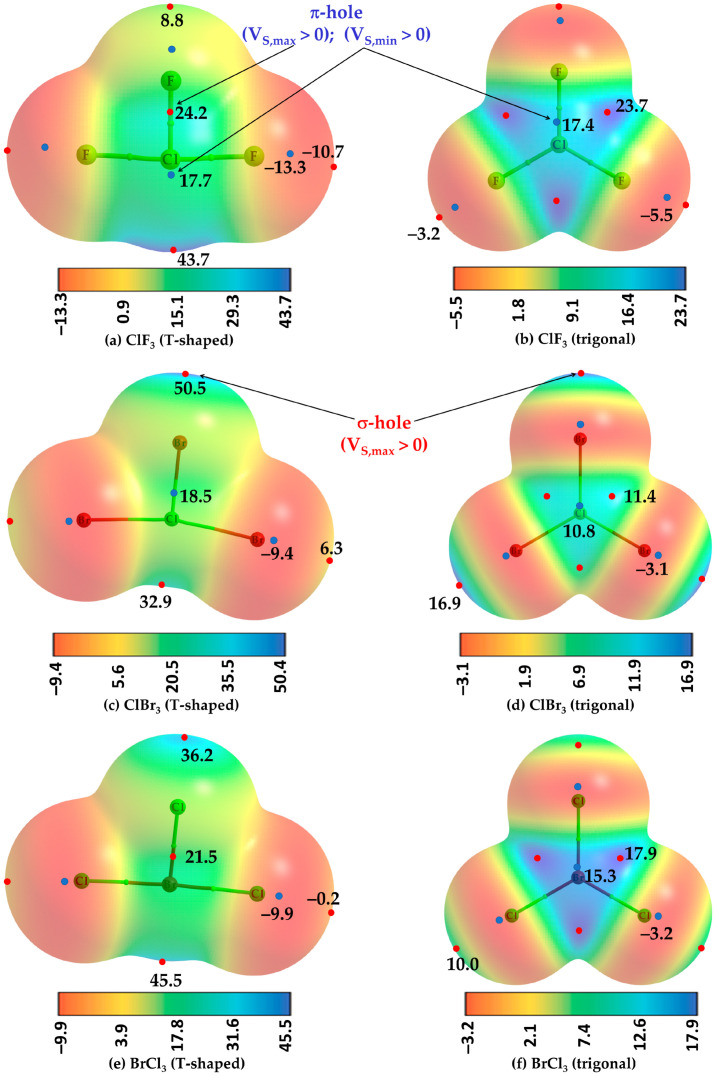
The [MP2/aug-cc-pVTZ] level potential on the electrostatic surface of (**a**,**b**) ClF_3_, (**c**,**d**) ClBr_3,_ and (**e**,**f**) BrCl_3_ molecules, illustrating the anisotropic nature of the charge density. The 0.001 a.u. (*electrons bohr^−^*^3^) isoelectronic density envelope was used to map the potential. The location of σ- and π-holes on the surfaces of the first two molecules (**a**–**d**) are indicated by arrows in black. The regions of local most maxima and minima of potential (V_S,max_ and V_S,min_) are marked by filled tiny circles in red and blue, respectively. Values are in kcal mol^−1^. The exact number of minima and maxima of potential are also on the opposite side of each of the three planar molecules (**b**,**d**,**f**), the side opposite to the viewer.

To illustrate the existence of a π-hole on halogen atoms in molecules such as ClF_3_, ClBr_3_, and BrCl_3_, two different geometries for each, planar (*C_2v_*) and T-shaped (*D_3h_*), were fully relaxed. The T-shaped configuration (left, Figure 1) is energetically favorable over the trigonal planar configuration (right, Figure 1) for each of the three cases. Both configurations represent a stationary point based on the positive sign of the eigenvalue of the Hessian matrix. The relative energy between the two configurations is ca. 16.44, 1.44, and 2.11 kcal mol^−1^ for ClF_3_, ClBr_3_, and BrCl_3_, respectively, suggesting that the planar geometry of ClF_3_ (point group, *D_3h_*) is unstable and lying far above the stable minimum.

Figure 1a–f shows the MESP graphs for both the configurations of ClF_3_, ClBr_3_, and BrCl_3_, each superimposed with its corresponding QTAIM’s molecular graph. The nature of the prominent electron density deficit is revealed on the surface of the central halogen atom for the most stable configuration (Figure 1a,c,e). Those regions are colored blue or cyan, green, or a combination of either of the two, where the extent of electron density deficiency is appreciable, characterized by the positive sign of the potential. From Figure 1a,c,e, it can be captured that the strength of the π-hole follows the following order: ClF_3_ (T-shaped) > BrCl_3_ (T-shaped) > ClBr_3_ (T-shaped), revealed by V_S,max_ > 0 for the former two and V_S,min_ > 0 for ClBr_3_. Conversely, the sign of potential is negative for a pair of σ-holes on the two halogen atoms responsible for the linearity of the Y-X-Y (X, Y = halogen) skeleton in ClF_3_ (Figure 1a) and BrCl_3_ (Figure 1e) and positive for ClBr_3_ (Figure 1c), signifying the presence of a lack of electron density deficiency on the surface of halogen in the latter. The stronger σ-holes are located along the outermost extensions of the remaining Cl–F/F–Cl bonds in ClF_3_ (Figure 1a), Cl–Br/Br–Cl bonds in ClBr_3_ (Figure 1c), and Cl–Br/Br–Cl bonds in BrCl_3_ (Figure 1e), characterized by V_S,max_ >> 0. The lateral surface portions of the two linearly arranged halogen atoms in the T-shaped geometries of the three molecules are electron-density rich, which may act as nucleophiles for HaBDs.

The electrostatic surfaces of the central Br and Cl atoms of the three planar molecules have six maxima and two minimum of potential (marked by tiny red and blue circles, respectively) orthogonal to the Br-Cl and Cl-Br/Cl-F bonding axes, respectively. A set of four extrema (three maxima and one minima) lying above the molecular plane is shown (Figure 1b,d,f), while the remaining set lying below the same plane is not shown (opposite to the viewer). Three of the four in a set appear in the junction regions (as potential maxima), each between a pair of adjacent halogen atoms, and the minimum of potential appears on the centroid surface area above the central halogen atom. They are each a representative electrophilic π-hole given the charge density is sufficiently deficient on the surface of these molecules, characterized by the positive sign of V_S,min_ or V_S,max_ (V_S,min_, V_S,max_ > 0).

The energy of the strongest π-hole, characterized by V_S,min_, is ca. 17.4 kcal mol^−1^ in ClF_3_ (Figure 1b), 10.8 in kcal mol^−1^ in ClBr_3_ (Figure 1d), and 15.3 kcal mol^−1^ BrCl_3_ (Figure 1f). The strength of the π-hole in these molecules described by V_S,max_ follows the order: ClF_3_ (23.7 kcal mol^−1^, Figure 1b) > BrCl_3_ (17.9 kcal mol^−1^, Figure 1f) > ClBr_3_ (11.4 kcal mol^−1^, Figure 1d). The trend is reasonable since F in ClF_3_ is highly electron-withdrawing than Cl and Br in ClBr_3_ and BrCl_3_, respectively, thereby creating a relatively strong electron density deficient region on the surface of Cl in ClF_3_. This may also be explained in terms of the increase in polarizability of the halogen derivative, which follows the order: Br > Cl > F. In all three cases, the lateral surface portions of the halogen derivative along the outer extensions Cl–F, Cl–Br, and Cl–Br covalent bonds are nucleophilic (characterized by the negative sign of V_S,min_). The σ-holes located at the outer extension of the Cl–F bonds in ClF_3_ are weakly nucleophilic; it is is more so for lateral portions of the same halogen atom. We did not observe a σ-hole on the surface the halogen along the F–Cl, Br–Cl, and Cl–Br covalent bond extensions in the geometries shown in Figure 1b,d,e, respectively. Nevertheless, the results above indicate that hypervalent halogen derivatives in halogenated molecules can not only be anisotropic but can host σ- and π-hole donors simultaneously.

### 2.2. Binary Complexes: Geometries and Bonding Features of π-Hole Halogen Bonds

To demonstrate the development of π-hole halogen bonding interaction between a pair of molecular entities, the T-shaped configuration of BrCl_3_ as the π-hole halogen bond donor and a series of Lewis bases as π-hole halogen bond acceptors were considered. The nucleophiles in the 32 HaBAs are either anions or neutral molecules. The Lewis base appears either on the entire electrostatic surface of the atom (as in the anions X^−^ (X = F, Cl, Br, CN) and OX^−^ (X = F, Cl, Br)) or around the bonding region, or on a small portion of an atom that constitutes the molecular entity. For instance, the nucleophile is concentrated around the bonding regions in F_2_, Cl_2_, Br_2_, ClF, BrF, and BrCl, among others, as well as on the lateral portions of the bonded atoms forming them. The outer portions on the sides of the halogen along the halogen–halogen bond extensions in these diatomic molecules are equipped with positive σ-holes. 

**Figure 2:**
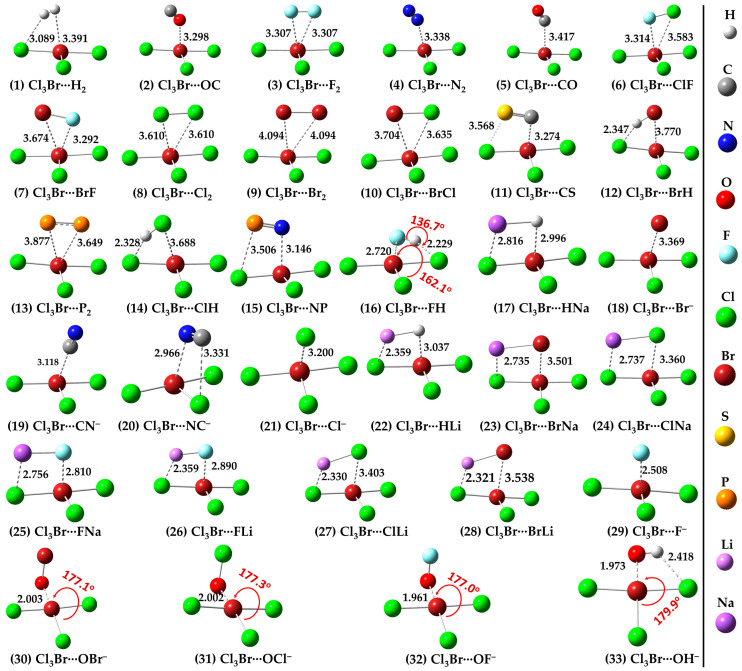
(1–33): [MP2/aug-cc-pVTZ] level fully relaxed geometries of 33 binary complexes of Cl_3_Br formed with 32 Lewis bases (H_2_, CO, N_2_, ClF, BrF, BrCl, PN, CS, X_2_ (X = P, F, Cl, Br), HX (X = F, Cl, Br), CN^−^, NC^−^, X^−^ (X = F, Cl, Br), LiX (X = H, F, Cl, Br), NaX (X = H, F, Cl, Br), OX^−^ (X = H, F, Cl, Br)). Selected bond distance/angles are in Å/degrees (°). Atom type is shown on the right panel. The solid and dotted lines between atomic basins represent covalent and non-covalent interactions, respectively.

Figure 2 (1–33) provides the theoretical evidence that the Lewis bases on the molecular entities above, characterized by negative electrostatic potentials, can show an aptness for attracting the electrophilic π-hole on the surface of Br in BrCl_3_. This causes the formation of π-hole halogen bonding interactions in the binary complexes investigated.

The weakest 1:1 binary complex in the series is observed between Cl_3_Br and H_2_ (Figure 2 (1)). Two types of intermolecular interactions may be speculated from the geometry of this complex. The outer nucleophilic part of the bonding region of the H_2_ molecule may be attractively engaged with the electrophilic π-hole on Br in Cl_3_Br, forming a Br(π)···H_2_ halogen bond. Similarly, an H atom in H_2_ may be engaged with the nucleophile on the nearest Cl atom in BrCl_3_, forming the H···Cl hydrogen bond. The occurrence of these two interaction types in the complex is not very surprising since the nucleophile on H_2_ is delocalized around the bonding region described by a belt-like negative potential, and the end portions of each of the two H atoms of the same molecule are reasonably charge density deficient and characterized by positive electrostatic potentials [74,75]. Clearly, the attraction between the interacting regions between the two molecules leading to the formation of the Cl_3_Br(π)···H_2_ dimer is primarily a result of Coulomb forces.

The intermolecular distances in Cl_3_Br···H_2_ are such that *r*(Br(π-hole)···H) = 3.391 Å, *r*(Br(π-hole)···(H_2_)_mid-point_) = 3.255 Å, and *r*(H···Cl) = 3.089 Å; the latter one is quasi-directional (∠H–H···Cl = 146.1°). They fail the distance-based feature for hydrogen [9] and halogen bonding recommended by IUPAC [8], given that neither of the intermolecular distances is less than the sum of the van der Waals (vdW) radii of respective atomic basins. For instance, the intermolecular distance for the Br···H contact is ca. 3.391 Å and is not less than vdW radii sum of H and Br atoms, 3.06 Å (*r*_vdW_(H) = 1.20 Å; *r*_vdW_(Br) = 1.86 Å [76]). Similarly, the intermolecular distance for the H···Cl hydrogen bond is 3.089 Å and is not less than van der Waals radii sum of H and Cl atomic basins, 3.020 (*r*_vdW_(H) = 1.20 Å; *r*_vdW_(Cl) = 1.82 Å [76]). As discussed by us and others [10,11,17,77], this type of failure of the IUPAC’s recommended feature is not surprising given that the vdW radii of atoms are not exact and have involved several approximations for their determinations and are associated with an error that can be as large as ±0.20 Å [10,78]. 

**Figure 3:**
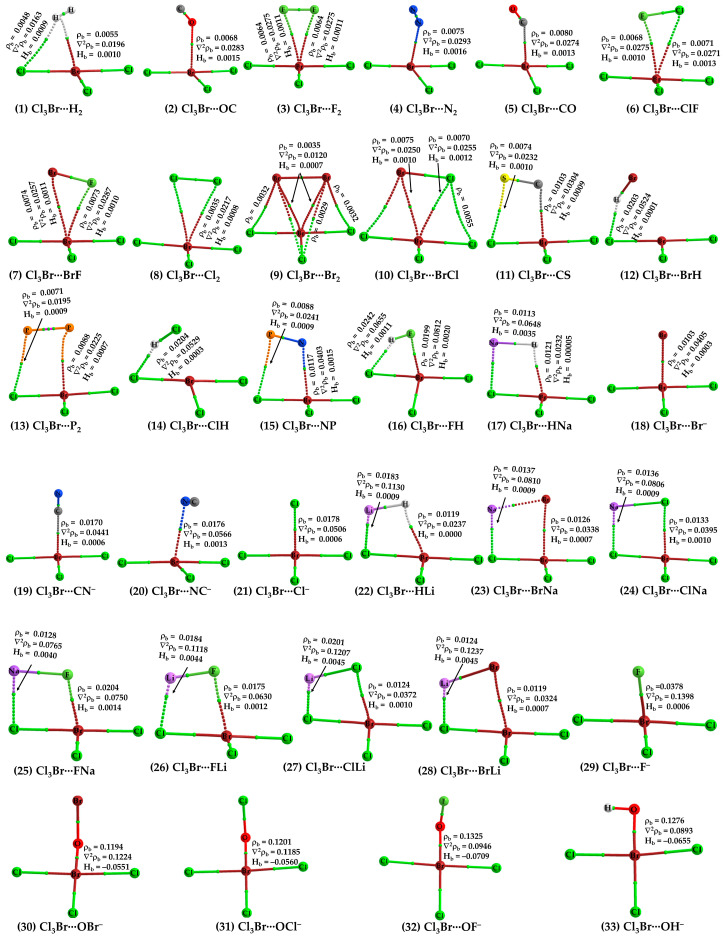
(1–33) [MP2/aug-cc-pVTZ] level QTAIM description of bonding topologies of all the 33 binary complexes of Cl_3_Br formed with 32 Lewis bases (H_2_, CO, N_2_, ClF, BrF, BrCl, PN, CS, X_2_ (X = P, F, Cl, Br), HX (X = F, Cl, Br), CN^−^, NC^−^, X^−^ (X = F, Cl, Br), LiX (X = H, F, Cl, Br), NaX (X = H, F, Cl, Br), OX^−^ (X = H, F, Cl, Br)). Each molecular graph comprises bond paths (solid and dotted lines in atom color) and bond critical points (tiny spheres in blue) between bonded atomic basins. Atom labeling is depicted in each case. Non-nuclear attractor (NNA) critical points (a pair of tiny spheres in green) are observed only between the two P atoms in Cl_3_Br(π-hole)···P_2_(13). The charge density (ρ_b_), the Laplacian of the charge density (∇^2^ρ_b_), and the total energy density (H_b_) values (in a.u.) are shown for selected bcps.

**Figure 4:**
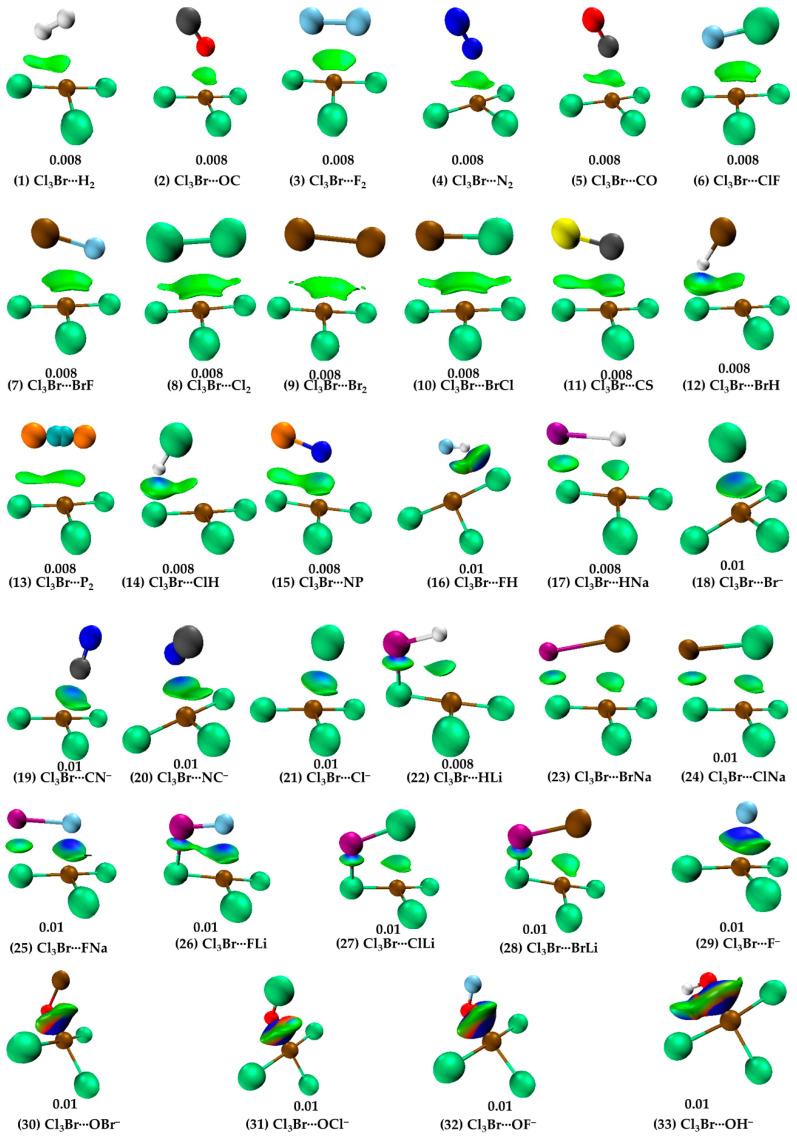
(1–33) [MP2/aug-cc-pVTZ] level IGM-*δg^inter^*-based isosurfaces, featuring the typical nature of intermolecular interactions exiting between interacting molecular entities in the 33 binary complexes investigated. Blue, cyan, and green isosurfaces indicate attractive interactions with decreasing strength, and reddish-like isosurfaces indicate repulsive interaction. Shown are isovalues, 0.008 and 0.01 a.u., that indicate weak-to-medium strength and strong interactions, respectively. The non-nuclear attractors (NNA) as overlapped balls in cyan are shown between the two P atoms in Cl_3_Br(π-hole)···P_2_(13).

The justification that the two monomer molecules in Cl_3_Br···H_2_ are noncovalently bonded is supported by the closeness of interacting regions that are having opposite charge capacities. Additionally, we have also observed the presence of charge-density-based bond-path (bp)/bond-critical-point (bcp) and isosurface (IGM-*δg^inter^*) topologies between interacting atomic basins, shown in Figure 3 (1) and Figure 4 (1), respectively. QTAIM gave two bond paths between Cl_3_Br and H_2_, one indicates the possibility of the Br(π-hole)···H halogen bond and the other implying the presence of an H···Cl hydrogen bond. The ρ_b_ (∇_2_ρ_b_ > 0) [H_b_ > 0] values are ca. 0.0048 (0.0163) [0.0009] and 0.0055 (0.0196) [0.0010] a.u. at the corresponding bcps, respectively (see Figure 3 (1)). (1 a.u. of ρ_b_ = *e*/*a*_0_^3^ = 6.748 eÅ^−3^ [79,80]; 1 a.u. of ∇_2_ρ_b_ = *e*/*a*_0_^5^ = 24.10 eÅ^−5^ [80]; 1 a.u. of H_b_ = 1 hartree = 627.5095 kcal mol^−1^ [81].)

Note that the bond path between Br(π-hole) and H_2_ is inwardly curved near H_2_, which indicates possible involvement of the bonding region in H_2_ with Br’s π-hole in developing the Br(π-hole)···(H–H)_mid-point_ closed-shell interaction. This is also confirmed by IGM-*δg^inter^*, revealing a flat-type distored dumbbell-shaped isosurface domain between the interacting moieties that suggests the presence of attractive interaction. The BSSE corrected interaction energy for the dimer is ca. −0.85 kcal mol^−1^ (see Table 1), leading to the rationale that Cl_3_Br···H_2_ is a vdW complex (vdW complexes usually have interaction energies < −1.0 kcal mol^−1^ [10,11]). NBO’s second-order perturbation theory revealed the H···Cl hydrogen bond is a characteristic of *np*(Cl) → σ*(H–H) charge transfer delocalization, with E^(2)^ = 0.95 kcal mol^−1^, where *np* and σ* refer to the p-type lone-pair orbital and σ-type anti-bonding orbital, respectively. A similar type of charge transfer delocalization (σ(H-H) → RY*(10) Br; E^(2)^ = 0.12 kcal mol^−1^) was yielded to describe the Cl_3_Br(π-hole)···H_2_ halogen bond, where RY* is an anti-bonding orbital called extra valence shell Rydberg orbital [82].

When N_2_ and CO were used as HaBAs, we did not observe any kind of sleep parallel arrangement between either of them and the π-hole on Br in BrCl_3_. The energy-minimized geometries of Cl_3_Br(π-hole)···OC, Cl_3_Br(π-hole)···CO, Cl_3_Br(π-hole)···N_2_ are shown in Figure 2 (2), (5) and (4), respectively. The spatial arrangement between the monomers in these dimers is logical since the central portion of N_2_/CO is charge density deficient and the nucleophile on them is mainly concentrated around the end portions of the N/C/O atoms [56,83]. The former causes steric repulsion between the bonding region in N_2_/CO and the electrophilic π-hole on Br in BrCl_3_, pushing them apart, aligning to an orientation so as to maximize the coulombic interaction with the electrophilic π-hole on the outer surface of the Br atom in BrCl_3_. Although QTAIM typifies the presence of the localized interaction between N/C/O in N_2_/CO and Br in BrCl_3_ (see Figure 3 (2), (5) and (4)), IGM-*δg^inter^*’s isosurface plot indicates a semi-elliptical- and triangle-shaped flat-type interaction between them that vivifies the possible presence of secondary interaction (see Figure 4 (2), (5), and (4)). The ρ_b_ (∇_2_ρ_b_ > 0) [H_b_ > 0] values are ca. 0.0068 (0.0283) [0.0015], 0.0075 (0.0293) [0.0016], and 0.0080 (0.0274) [0.0013] a.u. at the Cl_3_Br(π-hole)···OC, Cl_3_Br(π-hole)···N_2_, and Cl_3_Br(π-hole)···CO bcps, respectively, in which the trend in the ρ_b_ (or H_b_) values are in line with the interaction energies of the corresponding dimers (Cl_3_Br(π-hole)···OC < Cl_3_Br(π-hole)···N_2_ < Cl_3_Br(π-hole)···CO; see Table 1 for ΔE(BSSE) values). NBO’s second-order analysis has recognized Cl_3_Br(π-hole)···N_2_ to be the result of charge transfer delocalization from a π-bonding orbital of N_2_ to RY*(Br), (π(3) (N≡N) → RY*(6)Br) and back donation from the bonding Br-Cl orbital to RY*(N), (σ(Br-Cl) → RY*(2)N), with a corresponding E^(2)^ of 0.40 and 0.51 kcal mol^−1^, respectively. The charge transfer delocalizations π(3) CO → RY*(6)Br) (E^(2)^ = 0.43 kcal mol^−1^) and *np*(Cl) → π*(2) CO (and *np*(C) → RY*(5) Br) (E^(2)^ = 0.53 (0.10) kcal mol^−1^) cause the formation of the Cl_3_Br···CO and Cl_3_Br···OC dimers, respectively, where π*(2) and π(3) refer to the π* and π anti-double bond and triple bond orbitals, respectively.

The complexes of BrCl_3_ with CO, X_2_ (X = F, Cl, Br), N_2_, ClF, BrF, BrCl, CS, HX (X = Cl, Br), P_2_, or PN are weakly bonded. This is classified based on their BSSE corrected interaction energies, with the ΔE(BSSE) ranging from −1.31 to −4.84 kcal mol^−1^ (typical values for weak bonding range from −1.0 to −5.0 kcal mol^−1^). The π-hole halogen bonds are probably π_hole_^δ+^···lone-pair^δ−^-type in Cl_3_Br···X_2_ (Figure 2 (3), (8), and (9)), Cl_3_Br···P_2_ (Figure 2 (13)), Cl_3_Br···ClF (Figure 2 (6)), Cl_3_Br···BrF (Figure 2 (7)), and Cl_3_Br···BrCl (Figure 2 (10)), as well as in Cl_3_Br···CS (Figure 2 (11)), Cl_3_Br···OC/CO (Figure 2 (2) and (5)), Cl_3_Br···N_2_ (Figure 2 (2)), Cl_3_Br···XH (X = Cl, Br) (Figure 2 (12) and (14)), and Cl_3_Br···NP Figure 2 (15).

Secondary contacts are feasible in Cl_3_Br···CS and Cl_3_Br···NP. These are weak, not necessarily reliable indicators of actual interactions. The weak nature of the secondary interaction is revealed by the value of ρ_b_ at bcps, given that ρ_b_ is a measure of bond strength [84,85,86,87]. For example, the ρ_b_ are ca. 0.0088 and 0.0117 a.u for the P···Cl pnictogen bond and Br···N halogen bond in Cl_3_Br···NP, respectively. The corresponding values are ca. 0.0074 and 0.0103 a.u for the S···Cl chalcogen bond and Br···C halogen bond in Cl_3_Br···CS. Note that the pnictogen and chalcogen bonds in these dimers are not σ-hole centered but rather π-hole centered. In other words, P···Cl and S···Cl close contacts are π-hole pnictogen and chalcogen bonds, respectively.

**Figure 5:**
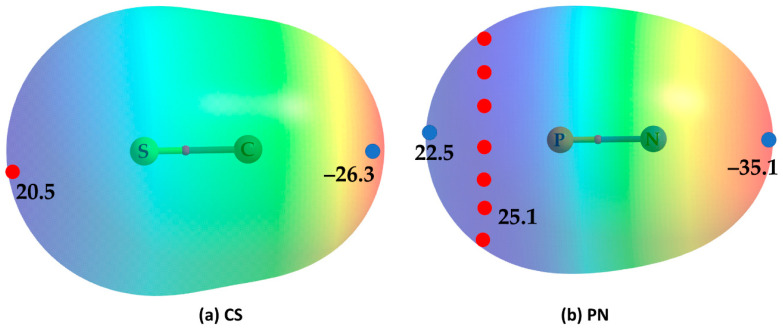
The [MP2/aug-cc-pVTZ] level potential on the electrostatic surface of (**a**) CS, (**b**) PN molecules, illustrates the anisotropic nature of the charge density. The 0.001 a.u. (*electrons bohr*^−3^) isoelectronic density envelope was used to map the potential. The regions of local most maxima and minima of potential (V_S,max_ and V_S,min_) are marked by filled tiny circles in red and blue, respectively. Values are in kcal mol^−1^. Most negative and positive regions are colored as red and blue, resepectively.

The perspective above may not be very surprising since the lateral portion of P in PN [43] and S in CS are positive. This may be inferred from the MESP plot shown in Figure 5a,b for these two molecules, respectively. As such, the V_S,min_ and V_S,max_ are −26.3 and 20.5 kcal mol^−1^ on the outermost extensions of the S=C and C=S bonds in CS, respectively, and the C-end along the outermost extension of the S=C bond is entirely negative. Similarly, it is conventionally understood that the electron density is concentrated around the P≡N bond region and that N is entirely negative. This is not the case; a ring of maxima is seen around the P atom (V_S,max_ = 25.1 kcal mol^−1^ each), and the negative electron density is far from the triple bond and is concentrated at the N terminus along the outermost extension of the P≡N triple bond. There is no maximum seen on the outermost extension of the P≡N bond, but there is an electron density deficit on P that appears probably to be due to a p-type orbital of the same atom and is described by a positive V_S,min_ (V_S,min_ = 22.5 kcal mol^−1^). The local electrophilic and nucleophilic pattern of this molecule may somehow resemble that of N_2_ [55,56], given that both molecules belong to the same pnictogen family. Nevertheless, the surface features of CS and PN are consistent with the bonding environment in the Cl_3_Br···CS and Cl_3_Br···NP dimers, in which Coulomb forces act between monomers that are presenter sites of opposite charge capacity. These also explain the reason why primary and secondary interactions are developed between the interacting monomers in the equilibrium structures of both dimers.

For Cl_3_Br···FH (Figure 2 (16)), the secondary interaction is stronger than the primary interaction. The hydrogen bond is the primary, and the halogen bond is secondary (ρ_b_ values 0.0242 vs. 0.019 a.u.). The importance of the secondary interactions towards the stability of this and other complexes is not only recognized by QTAIM (Figure 3 (2–15)) but also by IGM-*δg^inter^*’s isosurface topologies (Figure 4 (2–15)). NBO predicted that the strongest hyperconjugative interaction to occur from σ(F_2_) to RY*(7)Br/RY*(8)Br with E^(2)^ of 0.51/0.25 kcal mol^−1^ for Cl_3_Br···F_2_, σ(Cl_2_) to RY*(6)Br with E^(2)^ of 1.17 kcal mol^−1^ for Cl_3_Br···Cl_2_, and σ(Br_2_) to RY*(6)Br with E^(2)^ of 0.88 kcal mol^−1^ for Cl_3_Br···Br_2_. In the case of Cl_3_Br···P_2_, the charge transfer delocalization corresponds to π(2)P_2_/π(3)P_2_ → RY*(6)Br/RY*(5)Br with E^(2)^ of 0.23/0.13 kcal mol^−1^ and *np*(Cl) to σ*/π*(1/2/3) P_2_ with E^(2)^ < 1.8 kcal mol^−1^; the former is indicative of the presence of Br(π-hole)···π(P_2_) halogen bond.

The F_2_, Cl_2_, and Br_2_ molecules are symmetrically oriented on the top of the Br’s π-hole while forming dimers with BrCl_3_ (Figure 2 (3), (8), and (9), respectively). This is not the case for the dimers when ClF, BrF, and BrCl served as nucleophiles (Figure 2 (6), (7), and (10), respectively). Therefore, the topological properties of charge density (ρ_b_, ∇^2^ρ_b_ and H_b_) are identical for each pair of Br(π-hole)···F bcps in Cl_3_Br···F_2_, Br(π-hole)···Cl bcps in Cl_3_Br···Cl_2_ and Br(π-hole)···Br bcps in Cl_3_Br···Br_2_, but not in other three. In all of these dimers, the bond paths are developed between the terminal atoms of the dihalogen molecule and the Br’s π-hole, providing further indication that the attractive interaction in them is Coulombic in nature. The larger the negative end-portion of the halogen atom in the dihalogen molecule, the stronger the bend of that atom toward the Br’s π-hole (as in Cl_3_Br···FCl, Cl_3_Br···FBr and Cl_3_Br···ClBr). The secondary Br···Br interactions between the Br-ends of the Br_2_ molecule and the Cl atoms of the BrCl_3_ molecule are developed in the Cl_3_Br···Br_2_ dimer due to the larger size of the Br_2_ molecule. They are not only weak but also the forced consequence of the primary interaction.

Strong close contacts were observed for the complexes of BrCl_3_ with HF (Figure 2 (16)), LiX (X = H, F, Cl, Br) (Figure 2 (22), (26), (27), and (28), respectively), NaX (X = H, F, Cl, Br) (Figure 2 (17), (25), (24), and (23), respectively), and X^−^ (X = F, Cl, Br, CN) (Figure 2 (29), (21), (18), (19–20), respectively). The ΔE(BSSE) for these complexes varies between −6.53 and −20.49 kcal mol^−1^ (Table 1). For the former three sets with HF, LiH, and NaH as partner molecules, the large interaction energy mainly stems from two different types of intermolecular interactions. These interactions are the Br(π-hole)···H halogen bond and Li···Cl lithium bond in Cl_3_Br···HLi (Figure 2 (22)) and the Br(π-hole)···H halogen bond and Na···Cl sodium bond in Cl_3_Br···HNa (Figure 2 (17)). The geometrical stability of the Cl_3_Br···XLi (X = F, Cl, Br) and Cl_3_Br···XNa (X = F, Cl, Br) complexes are a result of additional Li···X and Na···X (X = F, Cl, Br) alkali bonds, respectively, accompanied by Br(π-hole)···X halogen bonds. In the case of the Cl_3_Br···HLi (Figure 2 (22)) and Cl_3_Br···HNa (Figure 2 (17)) complexes, hydrogen of LiH and NaH acts as nucleophiles in stabilizing the Br(π-hole)···H halogen bonds. The QTAIM results in Figure 3 ((22)–(28)) suggest that a specific type of interaction dominates between Cl_3_Br and alkali hydrides/halides when they are in close proximity. The electrostatic forces play a crucial role in stabilizing the dimers noted just above, evidence of the SAPT2+3(CCD) based analysis presented below. This perspective may also be transferable to the Cl_3_Br···X^−^ (X = F, C, Br, CN) anion–molecule complexes (see Figure 2 (30–33)), leading to the stability of charge-assisted π-hole halogen bonds. 

The results of QTAIM and IGM-*δg^inter^* justify the presence of both interaction types in the complexes involving LiX (X = H, F, Cl, Br) ((22), (26), (27), and (28) in Figure 3 and Figure 4, respectively), NaX (X = H, F, Cl, Br) ((17), (25), (24), and (23) in Figure 3 and Figure 4, respectively). The Br(π-hole)···X contacts in these complexes are slightly dispersive, evidenced by IGM-*δg^inter^*’s flat isosurface. The signatures such as ∇_2_ρ_b_ > 0 and H_b_ > 0, and a small ρ_b_ at the Li···X/Na···X, and Br(π-hole)···X bcps have provided further indication about the presence of closed-shell interactions in the dimers. Whereas we have not carried out NBO’s second order analysis for all the dimer systems, our results for the Cl_3_Br···HLi indicate that σ(Li–H) → RY*(3) Br (E^(2)^ = 1.16 kcal mol^−1^) and σ(Li–H) → σ*(Br–Cl) (E^(2)^ = 0.53 kcal mol^−1^), together with *np*(Cl) → σ*(Li–H) (E^(2)^ = 15.82 kcal mol^−1^), are the key hyperconjugative interactions that describe the Br(π-hole)···H and Li···Cl close-contacts.

The HF molecule showed different reactivity when placed near BrCl_3_ compared to HCl and HBr. The latter two molecules exhibit very similar intermolecular bonding patterns toward the formation of Br(π-hole)···X (X = Cl, Br) halogen bonds (see Figure 2 (14) and (12), respectively). Because of the strong electrophilic dispersion of the H atom due to the presence of the hemispherical σ-hole [88] and the negative hemisphere of F, the tiny HF molecule was unable to stand when its F-end was placed on the top of the Br’s π-hole. It flitted toward the most positive site on Br in BrCl_3_ during energy minimization, and in the fully relaxed geometry, the HF molecule is stable at a position shown in Figure 2 (16). In this geometry, both ends of the HF molecule are simultaneously engaged in bonding with BrCl_3_, forming two directional σ-hole-centered intermolecular contacts: one Br(σ-hole)···F halogen bond and one F–H···Cl hydrogen bond (Figure 2 (16)). The former bond is more so than the latter (∠Cl–Br···F = 162.1° and F–H···Cl = 136.7°). QTAIM and IGM-*δg^inter^* indicate the possible occurrence of both interactions in Cl_3_Br···HF (Figure 3 (16) vs. Figure 4 (16)). However, the Br(π-hole)···X bond path and critical point topologies of charge density missing in the molecular graphs of Cl_3_Br···ClH (Figure 3 (14)) and Cl_3_Br···BrH (Figure 3 (12)) are disqualified by IGM-*δg^inter^*; shown in Figure 4 (14) and (12), respectively. This limitation of the space partitioning approach has been reported previously, especially in the weak bonding regime [49,89,90,91].

The anion–molecule interactions observed in Cl_3_Br···X^−^ (X = F, Cl, Br) display very similar geometrical features as above. The Br(π-hole)···X^−^ contacts in them are charge-assisted π-hole halogen bonds. The strength of the interaction typified by the intermolecular bond distance increases with the increasing size of the halogen derivative. The trend in the bond distances (*r*(Br···F) = 2.508 Å; *r*(Br···Cl) = 3.200 Å; and *r*(Br···Br) = 3.369 Å) is in sharp agreement with the BSSE corrected interaction energies of the corresponding complexes(−20.49 kcal mol^−1^ for Cl_3_Br···F^−^; −12.75 kcal mol^−1^ for Cl_3_Br···Cl^−^; −11.33 kcal mol^−1^ for Cl_3_Br···Br^−^). NBO’s second-order analysis predicts multiple charge transfer delocalizations between the anion and molecule to explain the stability of the anion–molecule complexes. For Cl_3_Br···F^−^, they were *np*(4)F → RY*(2) Br, *np*(4)F → RY*(3) Br, *np*(3)F → σ*(Br–Cl), and *np*(4)F → σ*(Br–Cl), with a corresponding E^(2)^ of 2.73, 0.94, 0.43, and 1.84 kcal mol^−1^, respectively. For Cl_3_Br···Cl^−^, it was *np*(4)Cl → RY*(3) Br (E^(2)^ = 1.68 kcal mol^−1^) and for Cl_3_Br···Br^−^, it was *np*(4)Br → RY*(3) Br (E^(2)^ = 1.56 kcal mol^−1^).

Cl_3_Br···CN^−^ (Figure 2 (19)) and Cl_3_Br···NC^−^ (Figure 2 (20)) are the two dissimilar dimer geometries that resulted when the CN^−^ anion was used as the halogen bond acceptor. For Cl_3_Br···CN^−^, the C-end is directly involved in a Coulombic interaction with the π-hole on Br in BrCl_3_, while for Cl_3_Br···NC^−^, the C- and N-ends were both engaged with the electron density deficient regions on Cl and π-hole on Br in BrCl_3_, respectively. The Br(π-hole)···C and Br(π-hole)···N/Cl···C intermolecular distances are less the sum of the vdW radii of bonded atomic basins in Cl_3_Br···CN^−^ (Figure 2 (19)) and Cl_3_Br···NC^−^, respectively. The ΔE(BSSE) were −11.55 and −12.38 kcal mol^−1^ for the corresponding dimers, respectively, uncovering the strong nature of both dimers. The reliability of intermolecular contacts in these are validated by QTAIM- and IGM-*δg^inter^*-analyses, illustrated in (18–21) and (29) in Figure 3 and 4, respectively. The former predicts a localized closed-shell interaction between the interacting molecules in both the dimers of CN^−^ and misses a bond path that could describe the Cl···C close-contact in Cl_3_Br···NC^−^ (Figure 3 (20)). This was not the case with the results obtained from the IGM-*δg^inter^* analysis, which suggested a flat-type distorted-dumbbell-shaped interaction between the monomers causing the stability of the two dimers (Figure 4 (19) and (20)) and a possible involvement of the nucleophilic π-density of CN^−^.

The anion–molecule complexes formed between BrCl_3_ and XO^−^ (X = H, F, Cl, Br) (Figure 2 (30–32)) are not Br(π-hole) bonded. The anion rather engaged in a σ-hole interaction with Br in BrCl_3_. The Br(σ-hole)···O halogen bond is primarily responsible for the geometrical stability of these complexes and is potentially linear. The *r*(Br···O) (and ∠Cl–Br···X) are ca. 1.961Å (177.0°), 2.202Å (177.3°) and 2.203Å (177.1°) for Cl_3_Br···OF^−^, Cl_3_Br···OCl^−^ and Cl_3_Br···OBr^−^, respectively; the bond distances are markedly smaller than the sum of vdW radii of respective atomic basins. The contact distances are in line with the BSSE corrected interaction energies of −86.99, −81.46, and −80.99 kcal mol^−1^ for the corresponding complexes, respectively. The hydroxyl anion, OH^−^, on the other hand, shows an analogous bonding proficiency with the electrophilic σ-hole on Br in BrCl_3_, giving rise to the shortest bond distance, *r*(Br···O), of 1.973 Å. Not only is the intermolecular interaction in this complex highly directional (∠Cl–Br···O = 179.9°), but the BSSE-corrected interaction energy of −103.2 kcal mol^−1^ is the largest compared to all the anion–molecule complexes examined. The large interaction energy for this complex is not solely due to the Br···O halogen bond but also due in part to the H···Cl hydrogen bond.

The very large interaction energies for Cl_3_Br···OX^−^ (X = H, F, Cl, Br) suggest that the assembly of the Cl_3_Br molecule with the anions is a result of the formation of formal covalent bonds; a similar view that is also advanced in other studies for other complexes [33,92]. The argument counts on the results of the IGM-*δg^inter^* analysis (Figure 4 (30–33)), in which the isosurfaces representing the Br···O close-contacts are thickly shaped and colored blue, originating from the appreciable accumulation of charge density at the bond critical point region. QTAIM also gave the sign of H_b_ to be negative (H_b_ < 0) at the Br···O bcps of the Cl_3_Br···OX^−^, with appreciable character of ionicity (∇_2_ρ_b_ > 0), and the charge density at the corresponding bcps (ρ_b_ > 0.1 a.u.) was not very small (covalent bonds typically have ρ_b_ values ∼ 1.0 eÅ^−3^ [93]). This result is in contrast with that unveiled for the remaining 29 complexes (see (1–29) in Figure 2, Figure 3 and Figure 4.

Comparable interaction energies of anion–molecule complexes driven by σ-hole hydrogen and halogen bonds have been reported elsewhere [92,94]. It was shown [92] that the range of strongly halogen-bonded trihalides D-X···A^−^ and the analogous strongly hydrogen-bonded complexes D-H···A^−^ (D, X, A=F, Cl, Br, I) examined can be explained by the combined effect of electrostatic and covalent components stemming mainly from the HOMO–LUMO interaction between the occupied halide *np* atomic orbital (AO) and the D–H or D–X (X = halogen) σ* anti-bonding acceptor orbital. For the halogen-bonded complexes DX···A^−^, the contribution from the orbital interaction term was large, from 43% for F-I···F^−^ to 97% for I-F···F^−^. The larger covalent contribution is due to the lower orbital energy of the empty dihalogen σ* orbital (e.g., −0.7 eV for F-H and −6.2 eV for F-F), which results in stronger donor–acceptor orbital interactions with the halide *np* orbital.

It is worth noting that the proximity of the nucleophile in HaBA has induced geometric deformation into the Lewis acid. It is not just the Br-Cl bonding region that hosts the π-hole, but all three Br-Cl bonds in BrCl_3_ were distorted upon the formation of halogen bonds. For example, the Br-Cl bond giving BrCl_3_ its T-shape was elongated for all dimers, except for Cl_3_Br···HF and Cl_3_Br···OX^−^ (X = H, F, Cl, Br), for which it was contracted (all relative to the same bond length of 2.1353 Å for the isolated molecule). The elongation of the bond varied between 0.0014 Å (Cl_3_Br···H_2_) and 0.0186 Å (Cl_3_Br···CN^−^), while the contraction of the same bond was 0.0004 Å for Cl_3_Br···HF and varied between 0.2641 Å (Cl_3_Br···OBr^−^) and 0.3005 Å (Cl_3_Br···OH^−^) for all the four anion–molecule complexes.

Grabowski [95,96,97], as well as others [98,99], published a number of papers considering a few halogen bond donors (viz. BrF_3_) shown in Figure 1. In ref. [96], he labeled the σ-hole interactions as new types, even though such interactions formed by hypervalent halogen derivatives have been known for decades [99]. Similarly, in ref. [98], others have examined the complexes formed of H_2_Y (X = O, S, Se) with the hypervalent halogens XF_3_ and XF_5_ (X = Cl, Br, I). Their focus was to uncover the role of the electrophilic σ-hole on the X atom to form halogen bonding interactions with the lone-pair on the chalcogen atom in H_2_Y. Their results have shown that the interaction energies can be correlated with the properties of the X and Y atoms, that the formation of the complexes is dominated by electrostatics, and that the halogen bonds involving charge density donors H_2_S and H_2_Se possess some covalent character. Similar arguments have been provided by others [100].

### 2.3. SAPT-Based Energy Decomposition Analysis

To give substance to the notions developed above, SPAT2+3(CCD) level analysis was performed for all the binary complexes; the MP2 level optimized geometries were used. The results in Table 1 show that the energy contribution resulting from electrostatics (E_els_) is always attractive, meaning that Coulomb interactions play an important role in driving the monomers toward association. This, together with the induction (E_ind_) and dispersion (E_disp_) terms, is complementary with the exchange–repulsion energy component (E_exch_), giving rise to the net interaction energy, E[SPAT2+3(CCD)], for each dimer explored.

**Figure 6:**
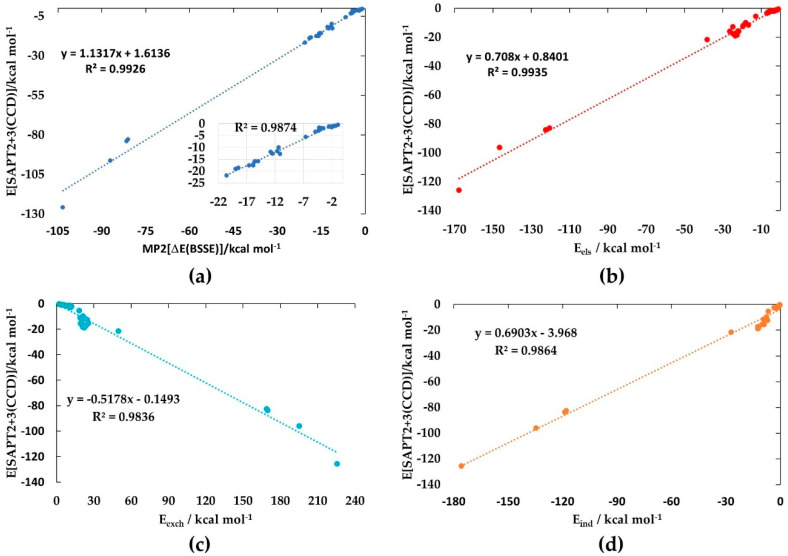
(**a**) Linear relationship between E[SPAT2+3(CCD)] and ΔE(BSSE) [MP2/aug-cc-pVTZ] for all the 33 binary complexes examined, described by the equation representing y. The insert marked by a black arrow in (**a**) corresponds to weakly to strongly bound complexes (ΔE(BSSE) < −22.0 kcal mol^−1^). The square of the regression coefficient, R^2^, is shown in each case. Plot of the dependence of E[SPAT2+3(CCD)] on (**b**) the electrostatic energy component (E_els_), (**c**) the exchange–repulsion energy component (E_exch_), and (**d**) the induction energy component (E_ind_) for the 33 complexes investigated (see Figure 2 for geometries and Table 1 for energies).

Figure 6a shows the correlation between E[SPAT2+3(CCD)] and ΔE(BSSE) [MP2/aug-cc-pVTZ], with the inset corresponding to the weakly to strongly bound complexes (ΔE(BSSE) < −22.0 kcal mol^−1^). The square of the regression coefficient, R^2^, is close to 0.9926. The slightly weaker regression occurs as a result of the anion-molecule complexes, dominated by large ΔE(BSSE) (ΔE(BSSE) > −80.99 kcal mol^−1^).

Figure 6b–d depicts the linear dependence of E[SPAT2+3(CCD)] on E_els_, E_ind_, and E_exch_, corresponding to R^2^ of 0.9935, 0.9836, and 0.9864, respectively. However, the dependence of E[SPAT2+3(CCD)] on E_disp_ was reasonably poor (R^2^ = 0.6198). This is because the E_disp_ could not be properly determined for most of the complexes. The large E_disp_ for the anion–molecule complexes (see Table 1 for E_disp_ values) gives the impression that the halogen-bonded interactions in them comprise some high degree of covalency. Nonetheless, Sengupta et al. [101] previously plotted each of the three attractive energy components against the [SAPT0/jun-cc-pVTZ] interaction energy for 37 small molecule complexes and observed that each component was linearly correlated. The correlation was strongest with the electrostatic component (R^2^ = 0.99), similar to what is observed in this study (R^2^ = 0.9935, Figure 6b). This has enabled the authors to demonstrate the overriding importance of electrostatics and the possibility of a predictive model in which the electrostatic energies can be considered as a match to the gas-phase total binding energies.

From the energies of the first 11 complexes in Table 1 (Figure 2 (1–13)) and Cl_3_Br···P_2_ (Figure 2 (13)), it may be seen that the dispersion term is dominant over the electrostatic component. This convinces us that, while dispersion certainly intensifies the strength of the interaction, the origin of the interaction is electrostatically driven, and polarization is an integral part of the Coulomb interaction [102,103,104,105,106,107,108].

For the remaining complexes, the scenario is not very different, meaning that the electrostatic contribution is the main driving force for their formation. The energy due to induction contributes appreciably to E[SPAT2+3(CCD)] for all complexes. However, for the first 15 complexes, the induction term dominates over the dispersion term, while for the remaining 18 complexes, the latter dominates over the former (Figure 2 (16–33)). For most complexes, E[SPAT2+3(CCD)] is comparable with ΔE(BSSE) [MP2/aug-cc-pVTZ]. The largest difference between them is found for anion–molecule complexes, Cl_3_Br···X^−^ (X = F, Cl, Br, OH), with deviations ranging from 2.01 to 22.81 kcal mol^−1^. It arises from the fact that SPAT2+3(CCD) relies on a “simple” pair model of each complex, and the charge polarization induced by the interacting molecule on the halide/hydronium anions could not be properly accounted for by SPAT2+3(CCD); a similar view is advanced elsewhere [109]. The deviational feature may be realized if E_els_ and E_ind_ are compared for each Cl_3_Br···OX^−^ (X = H, F, Cl, Br). Both the components are reasonably competitive with each other for these complexes, except for Cl_3_Br···OH^−^, in which case, the former (i.e., E_els_ = −167.51 kcal mol^−1^) is about 8.32 kcal mol^−1^ smaller in magnitude than the latter (E_ind_ = −175.83 kcal mol^−1^). These results enable us to conclude that the anion–molecule interactions are indeed dominated by electrostatics, yet reinforcing the conventional wisdom of the importance of the induction term to anion binding.

## 3. Materials and Methods

Geometry optimization and frequency calculations were performed for all 33 dimers using Gaussian 16 software [110] employing the MP2 method [111,112] in conjunction with Dunning’s type triple-ζ basis set aug-cc-pVTZ. The wave functions generated after geometry optimizations were used to calculate molecular graphs, bond critical point charge density properties, and molecular electrostatic surface potential graphs using AIMAll (version 19.10.12) [113]. Multiwfn (version 3.7) [114] and VMD (version 1.9.3) [115] software were used to calculate IGM-*δg^inter^* isosurfaces between interacting monomers, as well as for visualization and graph generations.

The dimers reported in the former section are identified as stationary points, evidenced by positive normal mode vibrational frequencies. Table S1 of Electronic Supplementary Information lists the fully-relaxed redundant internal coordinates of all dimers.

The σ- and π-hole features on the molecule’s electrostatic surface were characterized based on the signs of the local maximum and minimum of potential (symbolized by V_S,max_ and V_S,min_, respectively), which are the two key descriptors of the MESP model [55,116,117,118]. That is, when regions of a molecular surface are characterized by V_S,max_ < 0 (or V_S,min_ < 0) and V_S,max_ > 0 (or V_S,min_ > 0), we recognize them as electron density rich and electron density deficient regions, respectively. Accordingly, a σ-hole that is often located on the surface of atom A along the outermost extension opposite to an R–A covalent bond can be characterized by a positive or negative sign of V_S,max_ (V_S,max_ < 0, or V_S,max_ > 0). When the charge density is sufficiently deficient on the side of A opposite to the covalent bond extension (V_S,max_ > 0), we label the region as a positive σ-hole [15,17,40]; when the deficiency in the charge density is not appreciable, and characterized by a negative V_S,max_ (V_S,max_ < 0), we label the region on A as a negative σ-hole [47,53,119,120]. Chemical instances are known in which the outer extension of the covalent bond does not always feature a V_S,max_, but a V_S,min_. This is because the charge density is shifted from other parts of the molecule and accumulated around the outer extension of the bond (as on N in N_2_ [56], C/O in CO [121], and N in HCN [121], for example).

The characteristics of the π-hole can be understood based on positive or negative signs of the electrostatic potential. A region on a molecular entity can have either V_S,max_ > 0 (or V_S,min_ > 0) if the π-hole is electrophilic (electron density deficient) [52,122,123], or V_S,max_ < 0 (or V_S,min_ < 0) if nucleophilic (electron density rich) [47,55,56]. When such a situation is encountered, the former and latter characteristics signify the presence of positive and negative π-holes, respectively, and appear on the outer extensions around the covalently bonded atom (or molecular plane) orthogonal to the R-A bond axis [55]. It is worth noting that positive σ- and π-holes are not a region of electrostatic potential but correspond to a region of appreciable deficiency in electron density [17].

The reason for exploring QTAIM’s bond critical point features of charge density counts on IUPAC’s proposed feature put forward for hydrogen bonding [9]; this was also recommended for identifying halogen bonds [8], chalcogen bonds [7], pnictogen bonds [10,22], and tetrel bonds [11], among others [124,125]. The feature suggests that the charge density topology of the bond path and critical points could restore the shape of the molecule in terms of the molecular graph and that the sign and magnitude of the Laplacian of the charge density at the bond critical point, ∇^2^ρ_b_, should indicate the degree of charge density depletion (∇^2^ρ_b_ > 0) and concentration (∇^2^ρ_b_ < 0). Ourselves [10,11] and others [95] have recently suggested that the total energy density (H_b_) at the bond critical should also be scrutinized because it provides insight into the ionic/covalent (or mixed) nature of the chemical interaction under investigation. Considering that the bond critical point topology of QTAIM sometimes fails to appear in weak bonding regimes [49,89,90,126], an exploration of isosurfaces of charge density emerges from the application of reduced density gradient (RDG) [127] and independent gradient model (IGM-δg*^inter^*) [67,68] may be necessary, given these approaches have proven faithful in detecting weakly bonded interactions in chemical systems of diverse origin [10,11,56,109,128,129]. The theoretical details underlying these methodologies have been repeatedly discussed in several studies.

The uncorrected interaction energy, ΔE, of a dimer is calculated using the standard procedure given by Equation (1), where E_T_ is the electronic total energy of representative species. The ΔE was corrected upon incorporating the effect of basis set superposition error (BSSE), E(BSSE), accounted for by the counterpoise procedure of Boys and Bernardi [130], given by Equation (2).

ΔE (dimer) = E_T_ (dimer) − E_T_ (monomer1) − E_T_ (monomer2) (1)

ΔE (BSSE) = ΔE (dimer) + E(BSSE)
(2)

Given that there is much ongoing debate as to whether halogen bonding is an electrostatically driven phenomenon [24,44], a combined effect of electrostatics and (orbital) charge transfer [41,131], or electrostatics, exchange, polarization, and dispersion terms [40,132,133], we attempted to rationalize the dominant energy contribution to the interaction energy of the investigated dimers. We utilized the symmetry-adapted perturbation theory (SAPT) approach implemented in the PSI4 code [134], which has several variations, with zeroth-order (SAPT0) being the simplest [70]. The SAPT module can perform density-fitting evaluations of SAPT2, SAPT2+, SAPT2+(3), and SAPT2+3 energies only for closed-shell systems [69,70]; for methods such as SAPT2+, the many-body treatment of dispersion can be replaced by an improved method based on coupling cluster doubling (CCD) [71]. Whether CCD dispersion provides more accurate interaction energies tends to depend on the SAPT truncation and basis set employed due to the offsetting errors. We have employed the SAPT2+(CCD) level of theory, in conjunction with the basis set aug-cc-pVDZ, to provide our level of interpretation on the interaction energy of π-hole and σ-hole halogen bonded complexes, as reported elsewhere [135]. The NBO calculations were performed at the restricted Hartree-Fock level of theory, in conjunction with the basis set aug-cc-pVTZ, to estimate the second-order perturbative energy, E^(2)^, between the donor and acceptor orbitals responsible for non-covalent bond formation [73,136,137].

### Evidence of π-Hole Halogen Bonds Formed by Halogen Derivatives in Crystals: Yet to Be Fully Rationalized by Computational Approaches

**Figure 7:**
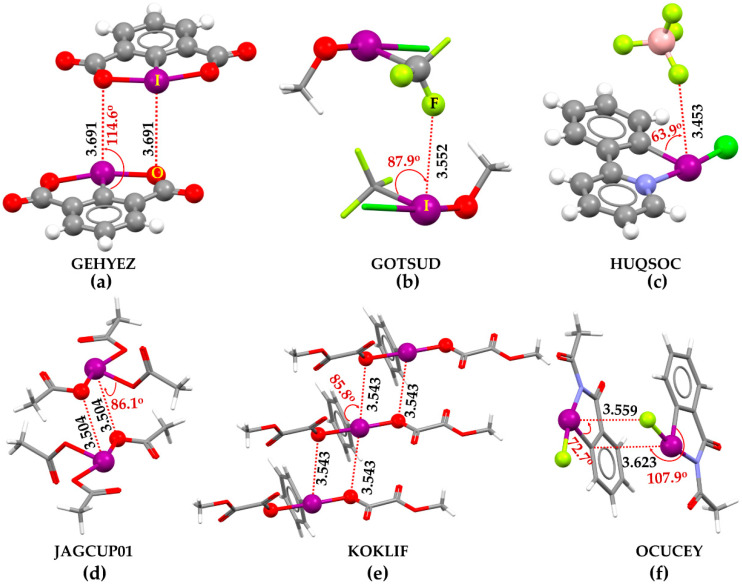
Illustrative crystallographic evidence of π-hole halogen bonds in crystals deposited to the Cambridge Structure Database (CSD): (**a**) iodosodilactone (C_8_H_3_IO_4_) [138]; (**b**) trifluoromethyl-methoxy-chloro-iodine(iii) (C_2_H_3_ClF_3_IO) [139]; (**c**) 5-Chloro-5H-5l^3^,6l^5^-pyrido[1,2-b][1,2]benziodazole tetrafluoroborate (C_11_H_8_ClIN^+^,BF_4_^−^) [140]; (**d**) iodine triacetate (C_6_H_9_IO_6_) [141]; (**e**) bis(Methyl oxalato)-phenyl-iodine (C_12_H_11_IO_8_) [142]; (**f**) 2-acetyl-1-fluoro-1,2-dihydro-3H-1,2-benziodazol-3-one (C_9_H_7_FINO_2_) [143]. The CSD reference code is shown for each case in uppercase letters. Selected halogen bond distances (and related angles) are in Å (and degrees), respectively. Labeling of selected atoms forming halogen bonds is shown in (**a**,**b**).

A search of vertical contacts of halogen in the CSD [1,2,3] has resulted in a number of crystals. In them, the higher derivative of halogen retains charge density-deficient regions that are probably electrophilic π-holes. Figure 7a–f provides insight into this in six such crystals, for example. The non-covalent interaction (dotted lines colored red) between the building blocks in these crystals may be readily speculated upon inspecting the intermolecular geometry. The π-holes on covalently bonded I develop I···O and I···F type π-hole halogen bonds, contributing to the adhesion between the building blocks to shape the crystals. They are not only predominantly non-linear but also directional in the sense that the nucleophile directs toward the π-hole on halogen. 

The intermolecular distances associated with the unique halogen bonds mentioned above are shorter than the vdW sum of the respective bonded atomic basins. That they are potentially non-linear, vertical interactions, evidence of the C–I···X (X = O, F, C) angles (∠C–I···O/F values between 63° and 115°). Our investigation shows that the halogen derivatives featuring π-holes in molecules can also form halogen bonds with charge density-rich regions hosted largely by O, F, C, and N atoms in neutral/anionicmolecular entities.

Some of the exemplary crystal systems in Figure 7 have been known since the 1980s. They have been called novel iodine(III) compounds. However, the I···O and I···F close contacts formed by covalently bonded iodine atoms in these systems were not assigned as halogen bonds. For example, the bis(methyloxalato)-phenyl-iodine (Figure 7e) and 2-acetyl-1-fluoro-1,2-dihydro-3H-1,2-benziodazol-3-one (Figure 7f) crystals have been known since 1989 and 2021, respectively, but the π-hole halogen bond in them was not appreciated. The authors of the study [143] have labeled them as secondary I···O bonding interactions developed between the λ^3^-iodine atom and the carbonyl oxygen of the acetyl group of the N-acetylbenziodazole skeleton. The I···N type close contacts were also observed in molecularly assembled crystals such as (((p-Nitrophenylsulfonyl)imino)iodo)-m-toluene [C_13_H_11_IN_2_O_4_S], and in crystal adducts such as [C_10_H_11_IN_3_O^+^,C_7_H_7_O_3_S^−^] with I···N bond distances of 3.667 [144], and 3.587 Å [145], respectively.

Previous studies [146] on hypervalent halogen atoms in neutral and anionic molecular entities containing bromine and iodine atoms have shown positive and negative σ-holes in their covalent bond extensions, respectively. Some of these crystal systems may also comprise vertical interactions between electrophilic iodine and interacting nucleophiles. However, the authors have suggested that crystallographic analysis can reveal close contacts, but calculated electrostatic potentials can provide insight into the extent of variations in positive potentials on the cationic surfaces and negative potentials on the anionic surfaces. Similarly, Wieske and Erdelyi [147] discussed the bonding features of hypervalent halogen (I) complexes. They argued that the halonium ions bound to two carbons, [C–X–C]^+^, do not possess halogen-bond donor character, as discussed by many research groups in the past. However, when nitrogen, sulfur, oxygen, or halogen-donor Lewis bases (D) are involved, hypervalent/hypercoordinate halonium ion [D···X···D]^+^ halogen-bond complexes are formed. With a view to this, it is unclear to us as to why the halonium ion bonded to the two carbons in [C-X-C]^+^ cannot be considered as halogen bond donors. It contradicts, not only to the very cationic character of halonium, but also to its versatile halogen bond donating ability. Our view is in line with Cavallo and coworkers [146], who explicitly discussed the halogen bond donating capacity of a range of iodonium and bromonium cations (viz. diphenyliodonium and dibenzo[b,d]iodonium and di-p-fluorophenylbromonium cations).

## 4. Conclusions

This study elucidates the potential existence of π-holes on halogen atoms within molecules, capable of forming π-hole halogen bonds with nucleophiles in another, representing a sister non-covalent interaction to σ-hole halogen bonds. π-hole halogen bonds exhibit many common features with σ-hole halogen bonds and other noncovalent interactions, with the primary distinction lying in their directional preference and spatial nature of occurrence. While σ-hole halogen bonds are highly directional axial interactions along the outermost extension of the halogen bond donor, π-hole halogen bonds are orthogonal interactions that occur perpendicular to the covalent bond axis or molecular plane. The intermolecular interaction distances associated with π-hole halogen bonds may not necessarily be less than the van der Waals sum of bonded atomic basins. Additionally, we observed that in some instances, particularly when atomic or diatomic anions were employed as nucleophiles, the π-hole of the halogen atom, such as that on Br in BrCl_3_, failed to form π-hole halogen bonds with interacting negative sites. This reaffirms the hypothesis of Politzer and coworkers that regions of positive or negative charge capacities on a molecular entity do not always attract regions of opposite charge capacities on another interacting molecular entity. Notably, in cases of anion–molecular complexes, the anion was unstable on the surface of the π-hole region but was able to adjust its spatial arrangement to maximize non-covalent interactions with the most positive sites, forming σ-hole halogen bonds with the interacting molecule. Furthermore, the interaction energy of π-hole halogen bonds was observed to be competitive with what has widely been reported for σ-hole halogen bonds, which can be van der Waals, weak, strong, very strong, and super-strong types depending on the nature of the nucleophile interacting with the halogen bond donor. The σ- and π-hole donors of halogen may be comparable in strength to aerogen, pnictogen, chalcogen, and tetrel bond donor entities that can also feature both σ- and π-holes, review by Zierkiewicz et al. [61]. We believe that this study is poised to significantly aid researchers across diverse fields of natural science in identifying and subsequently characterizing π-hole halogen bonds in chemical systems, both theoretically and experimentally.

## Figures and Tables

**Table 1:** [MP2/aug-cc-pVTZ] and [SAPT2+3(CCD)/aug-cc-pVDZ]-based interaction energies (ΔE/ΔE(BSSE) and E[SAPT2+3(CCD)], respectively) (kcal mol^−1^) of the binary complexes formed of Cl_3_Br with 32 Lewis bases. Included are also the BSSE energy (E(BSSE)) and dissected energy components (E_els_, E_exch_, E_ind_, and E_disp_) accounting for the SAPT2+3(CCD) interaction energies.

No.	Complex	ΔE	ΔE(BSSE)	E(BSSE)	E_els_	E_exch_	E_ind_	E_disp_	E[SAPT2+3(CCD)]
1	Cl_3_Br···H_2_	−1.49	−0.85	0.64	−0.99	2.13	−0.24	−1.46	−0.56
2	Cl_3_Br···OC	−2.15	−1.31	0.84	−1.30	2.75	−0.30	−2.19	−1.04
3	Cl_3_Br···F_2_	−2.3	−1.43	0.87	−1.34	3.00	−0.24	−2.36	−0.95
4	Cl_3_Br···N_2_	−2.66	−1.71	0.95	−1.69	3.42	−0.39	−2.47	−1.12
5	Cl_3_Br···CO	−2.96	−1.96	1.00	−2.64	4.43	−0.55	−2.84	−1.61
6	Cl_3_Br···ClF	−3.31	−2.23	1.08	−1.92	4.81	−0.53	−3.70	−1.34
7	Cl_3_Br···BrF	−4.00	−2.44	1.56	−2.36	5.89	−0.65	−4.24	−1.35
8	Cl_3_Br···Cl_2_	−4.72	−3.45	1.27	−3.21	7.19	−0.78	−5.19	−1.99
9	Cl_3_Br···Br_2_	−6.11	−3.93	2.18	−4.24	9.39	−1.03	−6.13	−2.01
10	Cl_3_Br···BrCl	−5.43	−3.69	1.74	−3.72	8.31	−0.91	−5.68	−2.00
11	Cl_3_Br···CS	−5.13	−3.9	1.23	−4.52	8.97	−1.38	−5.21	−2.13
12	Cl_3_Br···BrH ^a^	−5.96	−4.19	1.77	−6.21	12.15	−3.33	−5.27	−2.67
13	Cl_3_Br···P_2_	−5.43	−4.19	1.24	−5.16	10.75	−1.26	−6.02	−1.69
14	Cl_3_Br···ClH ^a^	−5.52	−4.31	1.21	−6.01	10.92	−3.33	−4.69	−3.11
15	Cl_3_Br···NP	−6.09	−4.84	1.25	−7.03	11.33	−2.27	−5.50	−3.47
16	Cl_3_Br···FH ^a^	−7.82	−6.53	1.29	−12.68	18.26	−6.48	−4.83	−5.74
17	Cl_3_Br···HNa	−12.32	−11.1	1.22	−24.79	24.67	−7.00	−5.71	−12.83
18	Cl_3_Br···Br-	−12.97	−11.33	1.64	−18.05	21.35	−7.57	−5.83	−10.10
19	Cl_3_Br···CN^–^	−12.93	−11.55	1.38	−16.57	19.07	−8.31	−5.86	−11.67
20	Cl_3_Br···NC^–^	−13.74	−12.38	1.36	−19.49	20.92	−8.32	−5.85	−12.75
21	Cl_3_Br···Cl^–^	−13.81	−12.75	1.06	−19.34	22.37	−9.30	−5.71	−11.98
22	Cl_3_Br···Hli	−16.46	−14.96	1.5	−26.30	25.57	−9.93	−5.33	−15.98
23	Cl_3_Br···BrNa	−17.43	−15.47	1.96	−21.94	20.44	−8.90	−5.45	−15.86
24	Cl_3_Br···ClNa	−17.1	−15.56	1.54	−21.61	19.50	−9.02	−4.94	−16.06
25	Cl_3_Br···FNa	−17.4	−15.8	1.6	−24.70	23.54	−12.06	−4.51	−17.73
26	Cl_3_Br···FLi	−18.41	−16.51	1.9	−22.79	21.89	−12.36	−4.36	−17.61
27	Cl_3_Br···ClLi	−20.26	−18.45	1.81	−22.58	21.10	−12.00	−5.17	−18.65
28	Cl_3_Br···BrLi	−21.22	−18.91	2.31	−23.47	22.62	−12.37	−5.87	−19.09
29	Cl_3_Br···F-	−21.97	−20.49	1.48	−38.05	49.97	−27.05	−6.74	−21.87
30	Cl_3_Br···OBr^– b^	−85.11	−80.99	4.12	−120.25	169.02	−117.88	−13.89	−83.00
31	Cl_3_Br···OCl^– b^	−84.92	−81.46	3.46	−122.33	169.95	−118.67	−13.03	−84.08
32	Cl_3_Br···OF^– b^	−90.42	−86.99	3.43	−146.33	195.19	−134.64	−10.61	−96.40
33	Cl_3_Br···OH^– b^	−106.67	−103.2	3.47	−167.51	225.56	−175.83	−8.23	−126.01

^a^ Comprises π- and σ-hole-centered halogen- and hydrogen-bonded interactions, respectively; ^b^ σ-hole-centered halogen-bonded interaction.

## Data Availability

Data are contained within the article and Table S1 (redundant internal coordinates of all dimers).

## Supplementary Materials

The supporting information can be downloaded at: https://www.mdpi.com/article/10.3390/ijms25094587/s1.
