# Supplementary material for: Halogen Bond via an Electrophilic π-Hole on Halogen in Molecules: Does It Exist?
Source: Int J Mol Sci. 2024 Apr 23;25(9):4587. doi: 10.3390/ijms25094587 (PMC11083155; doi:10.3390/ijms25094587)
Supplement: Supplementary file 1 [file ijms-25-04587-s001.zip › ijms-2918364-supplementary.pdf]

Halogen Bond Via an Electrophilic π-Hole on Halogen in Molecules: Does it Exist?

Pradeep R. Varadwaj[1,2*]

[1] Department of Chemical System Engineering, School of Engineering, The University of Tokyo 7-3-1, Tokyo 113-8656, Japan
[2] Molecular Sciences Institute, School of Chemistry, University of the Witwatersrand, Johannesburg 2050, South Africa
* Correspondence: pradeep@t.okayama-u.ac.jp (PRV)

Supplementary information

Table S1: Redundant internal coordinates of fully-optimized [MP2/aug-cc-pVTZ] geometries of all dimers, Br_3Cl···X (charge = 0/-1 and multiplicity = 1), where X refers to the molecules, or anions investigated, including H_2, CO, N_2, ClF, BrF, BrCl, PN, CS, X_2 (X = P, F, Cl, Br), HX (X = F, Cl, Br), CN^-, NC^-, X^- (X = F, Cl, Br), LiX (X = H, F, Cl, Br), NaX (X = H, F, Cl, Br), OX^- (X = H, F, Cl, Br).

(1) Br_3Cl···H_2
Br,0,0.3281041921,-0.2687555457,-0.3210637137
Cl,0,2.237149856,1.0632694449,-0.5304021328
Cl,0,0.0447614437,0.3237246535,1.7092121049
Cl,0,-1.56764964,-1.6390237462,-0.2664460872
H,0,-2.1561801716,2.0376041661,-0.4136708892
H,0,-2.3891956802,1.3360380273,-0.399144282

(2) Br_3Cl···CO
Br,0,0.455748913,-0.4044832995,-0.2948303827
Cl,0,2.4121827945,0.8580361944,-0.4943705566
Cl,0,0.1678615814,0.2232847113,1.7240933293
Cl,0,-1.4889502679,-1.70355754,-0.2504693178
C,0,-3.081898245,2.0507265684,-0.499259312
O,0,-1.967954776,1.8288503654,-0.4066787602

(3) $Br_3Cl\cdots F2$

Br,0,0.0452549083,-0.5679860052,-0.3541849832
Cl,0,2.3751635112,-0.3851514984,-0.4350610691
Cl,0,0.0110636762,-0.1393482806,1.735848407
Cl,0,-2.2837658518,-0.76226798,-0.4339357792
F,0,-0.9150296727,2.5918952145,-0.1762122473
F,0,0.4848944288,2.7051935497,-0.1764703283

(4) $Br_3Cl\cdots N_2$

Br,0,0.4826172005,-0.4117287044,-0.2919940065
Cl,0,2.4508212213,0.8337498268,-0.4829164715
Cl,0,0.1765072705,0.2376433231,1.7166997736
Cl,0,-1.4719405177,-1.6975797684,-0.2570557553
N,0,-3.0969422671,2.1245621077,-0.4890412381
N,0,-2.0440729076,1.7662102153,-0.4172073022

(5) $Br_3Cl\cdots OC$

Br,0,0.5071268896,-0.4128478661,-0.2872385542
Cl,0,2.4994526219,0.7930686883,-0.4762724608
Cl,0,0.2345730104,0.2027134507,1.7357338069
Cl,0,-1.4730820313,-1.6618394332,-0.2568247116
O,0,-3.1769253765,2.1314977406,-0.5282956195
C,0,-2.0941551141,1.8002644198,-0.4086174607

(6) $Br_3Cl\cdots ClF$

Br,0,0.0387440248,-0.6101801517,-0.3502096735
Cl,0,2.3755135056,-0.4641026722,-0.4193429529
Cl,0,-0.0025226291,-0.1499472893,1.7328323585
Cl,0,-2.2876694001,-0.7907508826,-0.4457403735
F,0,-0.9964000982,2.5289802765,-0.1082023323
Cl,0,0.5899155969,2.9283357193,-0.2493530264

(7) $Br_3Cl\cdots BrF$

Br,0,0.0596030893,-0.6164573516,-0.3459240173
Cl,0,2.3817974859,-0.4083999167,-0.4545281439
Cl,0,0.0307884277,-0.1443285408,1.7345384104
Cl,0,-2.2698155475,-0.8706929778,-0.4043762905
Br,0,-1.1101277666,2.8654294146,-0.2731092398
F,0,0.6253353112,2.6167843721,-0.096616719

(8) $Br_3Cl \cdots Cl_2$

Br,0,-1.0102738387,0.0000014383,-0.3566955286
Cl,0,-1.0318303373,-2.3385846139,-0.4403496994
Cl,0,-0.6223794187,-0.0000085223,1.740861506
Cl,0,-1.0320196447,2.3385851104,-0.440296149
Cl,0,2.4466593748,1.0004430468,-0.0706316029
Cl,0,2.4466878645,-1.0004294594,-0.0706585261

(9) $Br_3Cl \cdots Br_2$

Br,0,0.0549413176,-0.6723904402,-0.3440556344
Cl,0,2.3895757083,-0.5186193092,-0.4260227435
Cl,0,0.0139010187,-0.198314909,1.7350206438
Cl,0,-2.2735799824,-0.8986447019,-0.4314555673
Br,0,-1.3700824811,2.7726368874,-0.1880050039
Br,0,0.902825419,2.9576674729,-0.1854976947

(10) $Br_3Cl \cdots BrCl$

Br,0,0.0540858288,-0.6592337849,-0.3448900023
Cl,0,2.3846306404,-0.4839927597,-0.436253112
Cl,0,0.0179531233,-0.189642687,1.7354406873
Cl,0,-2.2750408695,-0.8954445371,-0.4211047485
Br,0,-1.3006969846,2.785258366,-0.2123361662
Cl,0,0.8366492616,2.8853904028,-0.1608726582

(11) $Br_3Cl \cdots CS$

Br,0,0.4499048948,-0.2289768513,-0.3441420394
Cl,0,2.237008883,1.2358914109,-0.5930463327
Cl,0,0.2174415021,0.2548214936,1.7186707517
Cl,0,-1.337072951,-1.7628437562,-0.2666914276
C,0,-1.8763701841,2.0742854487,-0.3092848992
S,0,-3.1939221448,1.2796792543,-0.4270210527

(12) $Br_3Cl \cdots BrH$

Br,0,0.2494815881,-0.5540838257,-0.3732851809
Cl,0,2.4884811559,-1.1621611152,-0.3966287052
Cl,0,0.2485222011,-0.2680533787,1.7386481906
Cl,0,-2.0327846058,0.0500256062,-0.5206073994
H,0,-1.1164293666,2.1934418793,-0.2442436171

Br,0,-0.1196899728,3.183165834,-0.043899288

(13) $Br_3Cl\cdots P_2$
Br,0,0.1294331399,-0.6857628956,-0.3257621592
Cl,0,2.4648803791,-0.6864836032,-0.3867045616
Cl,0,0.1113399806,-0.3722881492,1.7841895578
Cl,0,-2.2071475717,-0.6420087045,-0.4265765108
P,0,-1.3566947016,2.8940997061,-0.2633098354
P,0,0.5757697737,2.9347786465,-0.2218524909

(14) $Br_3Cl\cdots ClH$
Br,0,0.2693963346,-0.542602335,-0.3697431055
Cl,0,2.4986004587,-1.1817156384,-0.3782826412
Cl,0,0.2627983842,-0.235623839,1.7389855212
Cl,0,-2.0073225074,0.0862644205,-0.5329373124
H,0,-1.1083126461,2.2145593894,-0.2495106131
Cl,0,-0.197579024,3.1014530025,-0.0485278489

(15) $Br_3Cl\cdots NP$
Br,0,-0.1297244749,-0.6264251659,-0.3477273358
Cl,0,2.152292821,-0.0566398943,-0.4794646123
Cl,0,-0.1614905945,-0.3010863362,1.7567096311
Cl,0,-2.375835213,-1.2220177501,-0.391742757
N,0,-0.5589700398,2.4796264627,-0.08601454
P,0,0.7913085012,3.1688776838,-0.291776386

(16) $Br_3Cl\cdots FH$
Br,0,0.3436169025,-0.2614918691,-0.1425191977
Cl,0,1.0162080339,2.0258008695,-0.3553305348
Cl,0,-1.4704638244,0.1287103643,-1.1997730322
Cl,0,-0.167124567,-2.4992071408,0.1436411847
H,0,2.6138268793,0.9430283539,0.7593560461
F,0,2.7634514157,0.0673819422,1.0562815539

(17) $Br_3Cl\cdots HNa$
Br,0,0.2980522568,-0.4766795034,-0.3408887462
Cl,0,2.5495115494,-0.8642281604,-0.4155851007
Cl,0,0.2755916295,-0.1876861771,1.7582627246
Cl,0,-2.093143303,-0.1519816266,-0.4848757919

Na,0,-1.6239776476,2.6208878686,-0.3405914369
H,0,0.3115465149,2.502022599,-0.016337649

(18) $Br_3Cl\cdots Br^-$
Br,0,0.4515740722,-0.5359459224,-0.2532957623
Cl,0,2.3108651573,0.8630240967,-0.5587823611
Cl,0,0.2122337994,0.0380985792,1.7753818816
Cl,0,-1.2673124902,-2.1292600647,-0.1342165399
Br,0,-1.7217812188,2.0223178213,-0.5397040084

(19) $Br_3Cl\cdots CN^-$
Br,0,0.2430772619,-0.3738401562,-0.2637713182
Cl,0,1.9674816807,1.1891441068,-0.5747257993
Cl,0,-0.1994931833,0.324667513,1.6865011853
Cl,0,-1.3153226995,-2.1252331905,-0.1325895185
C,0,-2.343668989,2.2212849459,-0.0166440986
N,0,-1.8550840709,1.616833781,-0.9202854507

(20) $Br_3Cl\cdots NC^-$
Br,0,0.336334272,-0.4603206163,-0.2762176851
Cl,0,2.0450661771,1.1176357064,-0.5877281551
Cl,0,-0.0209372224,0.1489091891,1.7191700999
Cl,0,-1.2252307617,-2.2079665117,-0.1578987672
N,0,-2.7444118886,2.5421679782,-0.485326806
C,0,-1.8938305764,1.7124312543,-0.4335136864

(21) $Br_3Cl\cdots Cl^-$
Br,0,0.4261736555,-0.5060924038,-0.2669243344
Cl,0,2.2944537363,0.8855774256,-0.5618479233
Cl,0,0.1887149155,0.049045033,1.7661230434
Cl,0,-1.2911757201,-2.1043279357,-0.1642390605
Cl,0,-1.6325872672,1.9340323908,-0.4837285152

(22) $Br_3Cl\cdots HLi$
Br,0,0.3295663009,-0.4156550998,-0.3690678479
Cl,0,2.5461875229,-0.9202190109,-0.4127310435
Cl,0,0.2668033445,-0.1772648795,1.7344654344
Cl,0,-2.0575385697,0.0504810842,-0.5576642133

Li,0,-1.4769447854,2.3201323493,-0.2822136017

(23) Br_3Cl···BrNa
Br,0,0.2928369865,-0.5586263587,-0.3161646636
Cl,0,2.5216452454,-0.9600581589,-0.4241155198
Cl,0,0.2840039421,-0.2981256971,1.7886359378
Cl,0,-2.1157980154,-0.1913974867,-0.4265104191
Na,0,-1.9111099474,2.5355842078,-0.4009556383
Br,0,0.6460027888,2.9149584936,-0.060905697

(24) Br_3Cl···ClNa
Br,0,0.3238162257,-0.5291359153,-0.312879942
Cl,0,2.5613174337,-0.8915814456,-0.4155284515
Cl,0,0.3091215911,-0.2441583293,1.7881314606
Cl,0,-2.0923622987,-0.219545189,-0.4309154573
Na,0,-1.9036327298,2.5108464357,-0.4044592627
Cl,0,0.519320778,2.8159094436,-0.0643643471

(25) Br_3Cl···FNa
Br,0,0.3200883907,-0.4286172202,-0.336996742
Cl,0,2.5595040243,-0.842610719,-0.4111274936
Cl,0,0.2868659286,-0.1568947382,1.7630909142
Cl,0,-2.0872294578,-0.1069152164,-0.4981785734
Na,0,-1.6860391505,2.615273042,-0.3443302969
F,0,0.3243912647,2.3620998519,-0.0124738084

(26) Br_3Cl···FLi
Br,0,0.2959202939,-0.0524019312,-0.4327648208
Cl,0,2.3566914292,0.9054544471,-0.5050569463
Cl,0,0.1248532418,0.1055863454,1.6727440968
Cl,0,-1.8708859465,-1.1689253883,-0.5968751406
Li,0,-2.7940068382,0.9859503679,-0.3313384602
F,0,-1.6155821802,2.077193159,-0.0282237289

(27) Br_3Cl···ClLi
Br,0,0.3191699924,-0.4913871112,-0.3325504822
Cl,0,2.5235996161,-0.9963295598,-0.4072394552
Cl,0,0.2826302064,-0.2310029962,1.7709725132
Cl,0,-2.0800948063,-0.012077779,-0.4828681084

Li,0,-1.6525863039,2.2752330704,-0.362373865
Cl,0,0.3248622953,2.8978993758,-0.0259566025

(28) $Br_3Cl\cdots BrLi$
Br,0,0.2976041215,-0.5238228781,-0.3162127286
Cl,0,2.4942075729,-1.0461250466,-0.4099784226
Cl,0,0.2843683386,-0.2417923717,1.7850018317
Cl,0,-2.0995840934,-0.0155097335,-0.4441171702
Li,0,-1.6817557657,2.2663777422,-0.3813790713
Br,0,0.4227408262,3.0032072877,-0.073330439

(29) $Br_3Cl\cdots F^-$
Br,0,0.3432965005,-0.3674692965,-0.2935663915
Cl,0,2.3643869916,0.8593374377,-0.5548572475
Cl,0,0.1371412397,0.2075613876,1.7412827088
Cl,0,-1.4506570424,-1.8678376229,-0.259525033
F,0,-1.4085883694,1.4266426041,-0.3439508267

(30) $Br_3Cl\cdots OBr^-$
Br,0,-0.0838855129,0.0989681057,0.0799482591
Cl,0,1.4866653506,-0.1537678992,-1.6940176283
Cl,0,-1.6344196425,0.4857108591,1.8473571703
Cl,0,-1.2031900744,-1.9308245605,-0.539990902
Br,0,0.2439938886,3.2027439619,-0.1915601435
O,0,0.9084169904,1.7395045329,0.6582472444

(31) $Br_3Cl\cdots OCl^-$
Br,0,-0.0874876748,0.1180722521,0.0734057483
Cl,0,1.4858713792,-0.1387387131,-1.6977721351
Cl,0,-1.6370813417,0.5015871928,1.8426499058
Cl,0,-1.199203957,-1.9190481243,-0.5387896929
Cl,0,0.2608729705,3.1133388269,-0.1609558533
O,0,0.8946096238,1.7671235655,0.6414460272

(32) $Br_3Cl\cdots OF^-$
Br,0,-0.1022936047,0.183084972,0.0581153917
Cl,0,1.510795194,-0.1390922927,-1.6697742817
Cl,0,-1.6895507656,0.5920823427,1.7915499243

Cl,0,-1.2700828325,-1.8237864804,-0.5976938919
F,0,0.3657324876,2.8722214986,-0.0771555658
O,0,0.9029805212,1.7578249598,0.6549424235

(33) $Br_3Cl\cdots OH^-$
Br,0,0.0139631542,-0.1958234644,0.0000726749
Cl,0,2.3421180134,-0.4400981872,0.0000246785
Cl,0,0.1160125525,2.2378358195,-0.0000642761
Cl,0,-2.3987363023,-0.2420934872,0.0001363548
H,0,-1.0364707011,-2.2397332088,0.000181244
O,0,-0.0637727168,-2.1677154717,0.0001833238
